# Self-Perceived Health Status of Patients with Newly Diagnosed Diabetes in Spain: Associated Factors and Sex Differences

**DOI:** 10.3390/jcm14196770

**Published:** 2025-09-25

**Authors:** Pilar Vich-Pérez, Belén Taulero-Escalera, Paula Regueiro-Toribio, Isabel Prieto-Checa, Victoria García-Espinosa, Laura Villanova-Cuadra, Ignacio Sevilla-Machuca, Julia Timoner-García, Mario Martínez-Grandmontagne, Tania Abós-Pueyo, Cristina Álvarez-Hernández-Cañizares, Germán Reviriego-Jaén, Alberto Serrano-López-Hazas, Inés Gala-Molina, Mar Sanz-Pascual, María José Guereña-Tomás, Ana Isabel González-González, Miguel Angel Salinero-Fort, on behalf of the LADA-PC Consortium

**Affiliations:** 1 Los Alpes Health Center, 28022 Madrid, Spain; pilar.vich@salud.madrid.org (P.V.-P.); isabel.prietocheca@salud.madrid.org (I.P.-C.); victoria.garciaes@salud.madrid.org (V.G.-E.); ignacio.sevillama@salud.madrid.org (I.S.-M.); mariajose.guerena@salud.madrid.org (M.J.G.-T.); 2 Foundation for Biosanitary Research and Innovation in Primary Care, 28003 Madrid, Spain; belen.taulero@salud.madrid.org (B.T.-E.); aisabel.gonzalez@salud.madrid.org (A.I.G.-G.); 3 The Hospital La Paz Institute for Health Research (IdiPAZ), 28046 Madrid, Spain; 4 Mar Báltico Health Center, 28033 Madrid, Spain; paula.regueiro@salud.madrid.org; 5 Dr. Cirajas Health Center, 28017 Madrid, Spain; laura.villanova@salud.madrid.org (L.V.-C.); julia.timoner@salud.madrid.org (J.T.-G.); 6 Sanchinarro Health Center, 28050 Madrid, Spain; mario.martinez@salud.madrid.org; 7 Estrecho de Corea Health Center, 28027 Madrid, Spain; mariatania.abos@salud.madrid.org; 8 Canillejas Health Center, 28022 Madrid, Spain; calvarezh@salud.madrid.org; 9 Barajas Health Center, 28042 Madrid, Spain; german.reviriego@salud.madrid.org; 10 García Noblejas Health Center, 28037 Madrid, Spain; alberto.serrano@salud.madrid.org; 11 Benita de Ávila Health Center, 28043 Madrid, Spain; ines.gala@salud.madrid.org; 12 Aquitania Health Center, 28032 Madrid, Spain; mspascual@salud.madrid.org; 13 Research Network on Chronic Diseases, Primary Care and Health Promotion (RICAPPS), Carlos III Health Institute (Instituto de Salud Carlos III), Av. De Monforte de Lemos, 5, 28029 Madrid, Spain; 14 Department of Biomedicine, Universidad Alfonso X El Sabio, 28691 Villanueva de la Cañada, Spain

**Keywords:** self-perceived health, patient-reported outcome measures, social determinants of health, diabetes mellitus, exercise, Mediterranean diet, obesity, morbid

## Abstract

**Background/Objectives**: Multiple studies indicate that self-perceived health accurately reflects a person’s overall health, and that poor self-perceived health is associated with chronic diseases, the use of health services, increased health expenditure, and mortality. There is little research on this topic in people with newly diagnosed diabetes mellitus (DM). The objectives of this study were to analyse self-perceived health in adult patients with newly diagnosed DM (mostly T2DM, but also T1DM), identify associated characteristics, including a Mediterranean diet and physical activity, and examine differences by sex. **Methods**: This is a cross-sectional study of 796 patients. Participants were evaluated through physical examination, electronic medical records, self-perceived health assessment, lifestyle, personal and family history, and laboratory parameters. A multivariate analysis was performed on the total sample and on sex subgroups to identify factors associated with self-perceived health. **Results**: In total, 25.8% of participants reported poor/fair health (33.4% women, 20.2% men). The variables associated with poorer self-reported health were being female, a lack of family support, morbid obesity, low physical activity, and anxiety/depression. In women: morbid obesity, cancer, and antiplatelet therapy. In men: lack of family support, age < 60, anxiety/depression, low physical activity, and previous treatment with ACEIs/ARBs. **Conclusions**: Poor self-perceived health is common in patients newly diagnosed with diabetes, similar to the general population. The factors found explained 24.2% of the variance and showed different associations by sex. The cross-sectional design did not allow for inferences of causality. These findings could suggest personalised interventions to address psychosocial and lifestyle factors at the onset of diabetes.

## 1. Introduction

Perceived health status is a commonly used health indicator in scientific studies. In 1982, Mossey and Shapiro reported that poor perceptions of health were associated with reduced life expectancy [1]. Subsequent studies have further established that self-perceived health is a reliable reflection of an individual’s overall health and is strongly associated with chronic diseases, the use of health services, increased health expenditures, and mortality [2,3,4,5,6,7,8,9].

Perceived health status is defined as an individual’s subjective assessment of their health over the past 12 months [10]. In most studies, factors such as being female, being older, being obese, having a sedentary lifestyle, having low economic and educational levels, having poor social or family support, having depression, and having chronic diseases or disabilities were associated with lower health perception scores. Given that some patients have a negative perception of their health, positive changes in certain conditions, such as increased physical activity and a healthier diet, can help them achieve better health outcomes [11,12,13,14,15,16].

There are few studies on health perceptions in people with newly diagnosed diabetes mellitus (DM). Classically, insulin treatment and disease complications are associated with poorer health perceptions in patients with DM [17,18]. However, fewer complications and insulin treatments are expected in the early stages of the disease.

The primary objective of this study was to analyse the self-perceived health of adult people with newly diagnosed DM (mostly T2DM, but also T1DM) and to identify the characteristics most strongly associated with perceived poor or fair health status, both in the total sample and when stratified by sex. In addition, the study aimed to assess whether adherence to the Mediterranean diet and engagement in physical activity are associated with self-perceived health.

## 2. Materials and Methods

This was a cross-sectional descriptive study conducted in primary care settings within the Community of Madrid (Spain), involving 796 patients with newly diagnosed adult-onset diabetes from 12 health centers. The design of this study has been published previously [19]. Briefly, the researchers interviewed patients about their self-perceived health, lifestyle, and personal and family history. Health data were collected through physical examinations, electronic medical records (EMRs), and laboratory parameters. The inclusion criteria were: recent diagnosis of diabetes in individuals over 30 years of age, diagnosed at least 6 months and no more than 4 years prior. The exclusion criteria were gestational diabetes, homebound status, and inability to complete the interview or physical examination.

During the enrolment visit, participants were checked to ensure that they met the American Diabetes Association (ADA) diagnostic criteria for DM [20] and were checked for blood and urine tests.

At a subsequent visit, the remaining study variables were collected, and the participants were informed of their laboratory results. As recruitment is still ongoing, there are subjects whose data collection sheets have yet to be fully completed. A study flowchart is shown in Supplementary Figure S1.

### 2.1. Definition of Variables

Anthropometric parameters, such as weight, height, waist circumference, and body mass index (BMI), were measured via standard methods [21].

Self-perceived health: Self-perceived health was assessed by asking the following question: “How would you rate your health?” The response options were “excellent”, “very good”, “good”, “fair”, and “poor”. For analysis purposes, responses were dichotomized as “positive self-perceived health” (excellent, very good, and good) or “negative self-perceived health” (fair and poor), following previous research on this topic.

Physical activity: A reduced scale of the International Physical Activity Questionnaire (IPAQ) was used, with the following categories: low (not performing any physical activity or insufficient activity performed to meet IPAQ category 2 or 3), moderate (≥three sessions/week of vigorous physical activity for at least 20 min/day; ≥five sessions/week of moderate physical activity and/or walking for at least 30 min/day; or ≥five sessions/week of any combination of walking and/or moderate and/or vigorous physical activity), and high physical activity (≥three sessions/week of vigorous physical activity for at least 60 min/day, or ≥seven sessions/week of any combination of walking and/or moderate and/or vigorous physical activity).

Mediterranean diet: A modified version of the Mediterranean Diet Adherence Screener (MEDAS) questionnaire was used to analyse adherence to the Mediterranean diet. Scores of 11–14 indicate high adherence. The modification involved adjusting wine consumption from two drinks per week instead of seven, as in the original MEDAS [22], to improve screening for alcohol consumption, since the questionnaire categorized risk on the basis of the type of drink and grams/week, not just the number of alcohol units.

Metabolic syndrome (MS): Defined according to the National Cholesterol Education Program Adult Treatment Panel III as the presence of at least three of the following components: (1) abdominal circumference ≥ 102 cm in men and ≥88 cm in women; (2) triglycerides ≥ 150 mg/dL; (3) blood pressure ≥ 130/85 mm/Hg or established hypertension; (4) HDL cholesterol < 40 mg/dL in men and <50 mg/dL in women; and (5) fasting plasma glucose 110 to 126 mg/dL (6.11–6.99 mmol/L) or DM diagnosis [23].

Polypharmacy is defined as the regular use of five or more medications per day, as reported in most studies [24]. The remaining variables are specified and defined in Supplementary Table S1.

### 2.2. Statistical Analysis

Descriptive analyses were performed for all the variables included in the present analysis. Quantitative variables were summarized using the mean and standard deviation or the median and interquartile range, depending on the type of distribution. Qualitative variables are presented as absolute or relative frequencies (%). The Student’s *t* test or the nonparametric Mann–Whitney U test was used to compare subgroups of quantitative variables, depending on whether they followed a normal distribution. Normality was assessed based on the Central Limit Theorem and visual inspection of Q–Q plots. The chi-square test or z test for the comparison of proportions was used to compare qualitative variables.

Univariate analysis was conducted to determine the odds ratio for poor/fair health perception for each variable. Multivariate analysis was performed via binary logistic regression to identify the variables with the most significant magnitude of association with poor/fair health perception and to examine the associations of physical activity and adherence to the Mediterranean diet with self-reported health status. The models were adjusted for sociodemographic characteristics (sex and education), psychosocial covariates (social support), lifestyle factors (BMI, Mediterranean diet, and physical activity), comorbidities (CKD, retinopathy, neuropathy, hypertension, CVD, atrial fibrillation, respiratory disease, psychotic disorders, mood disorders, and cancer), and therapies (DPP-4 inhibitors, SGLT2 inhibitors, antihypertensive therapies, antiplatelet drugs, psychiatric and mood disorder medications, analgesics, corticosteroids, anticancer medication, immunomodulators, and polypharmacy). Variables were introduced into the model stepwise on the basis of statistical significance in the univariate analysis (*p* < 0.05), and adjustment variables were considered clinically relevant. Fasting plasma glucose (FPG), HbA1c, total cholesterol, and LDL cholesterol were dichotomized using standard control thresholds (FPG < 100 mg/dL, HbA1c < 7%, total cholesterol < 200 mg/dL, and LDL cholesterol < 100 mg/dL) and excluded from the multivariate analysis due to non-significant *p*-values and because, in newly diagnosed diabetes, patient priorities typically focus on self-management education rather than lipid or glucose control.

To assess potential collinearity among the multiple variables included in the logistic regression models, we used the STATA command “regress vif”. This analysis showed that none of the variables exceeded a variance inflation factor (VIF) value of 5, indicating an absence of significant multicollinearity.

Supplementary Table S2 presents the absolute and relative frequencies of missing data for all variables. Supplementary Figures S2 and S3 display the missing data analysis and the assessment of missingness patterns, respectively.

The variable “number of tobacco pack-years” was excluded from the analysis, due to a high proportion of missing data (46%), in order to minimize potential bias and preserve analytical validity. This level of missingness was likely attributable to the complexity of its calculation under routine clinical conditions and the workload of general practitioners. Conversely, the variable “tobacco use” was retained, with complete data availability.

Missing values were imputed using a non-iterative, single-pass mean substitution method. This approach was considered appropriate, as the variables with missing data were used exclusively for descriptive purposes, specifically to characterize the distribution of relative frequencies across categories of self-perceived health, and not for inferential analyses. Additionally, their distributions were approximately normal, as confirmed by Q–Q plots.

The statistical analysis was performed using SPSS for Windows, version 26.0 (IBM SPSS, Armonk, NY, USA: IBM Corp.) and STATA version 16 (StataCorp, College Station, TX, USA). A two-tailed *p*-value < 0.05 was considered to indicate statistical significance.

## 3. Results

The study sample included 455 men (57.2%) and 341 women. The mean age was 61.9 years (SD = 11.0), and the mean age at the diagnosis of diabetes mellitus was 59.3 years (SD = 11.0). Overall, 25.8% of participants (95% confidence interval (CI), 22.8–28.8) reported negative self-perceived health (poor/fair), with a significantly higher proportion in women (33.4%; 95% CI, 28.4–38.4) than in men (20.2%; 95% CI, 16.5–23.9) (*p* < 0.01) (Table 1). Supplementary Table S3 presents the distribution of quantitative variables stratified by self-perceived health status. Statistically significant differences were observed in body mass index (BMI), total cholesterol, LDL cholesterol, and the number of medications taken per day.

**Figure 1:**
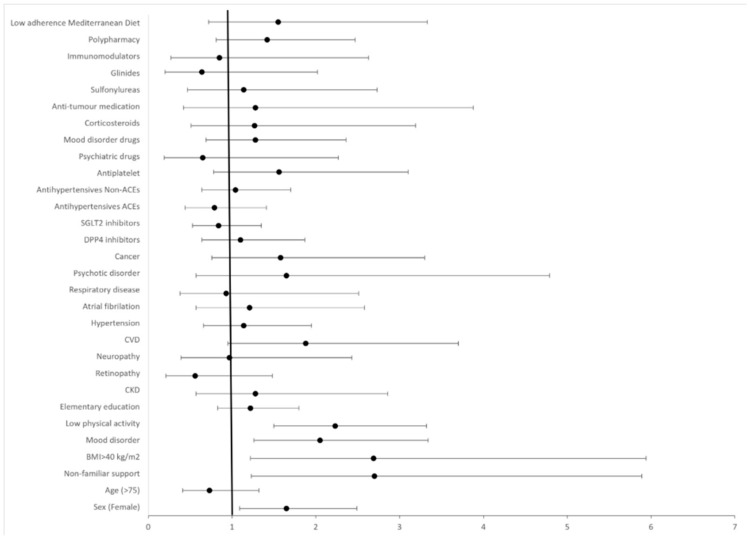
Forest plot displaying adjusted odds ratios (aORs) and 95% confidence intervals (CIs) for variables examined as potential factors associated with poor/fair self-reported health. *p*-values: female sex (0.014), age > 75 years (0.381), elementary education (0.348), non-familial support (0.020), BMI > 40 Kg/m^2^ (<0.001), CKD (0.465), retinopathy (0.212), neuropathy (0.876), hypertension (0.624), CVD (0.060), atrial fibrillation (0.686), respiratory disease (0.708), mood disorder (0.003), psychotic disorder (0.405), cancer (0.200), DPP4-i (0.533), SGLT2-i (0.405), antihypertensives—ACEIs (0.343), antihypertensives—non-ACEIs (0.876), antiplatelet drugs (0.233), psychiatric drugs (0.441), mood disorder drugs (0.386), glinides (0.363), sulfonylureas (0.794), anticancer medication (0.465), immunomodulators (0.689), corticosteroids (0.522), polypharmacy (0.253), adherence to Mediterranean diet (0.269), and low physical activity (<0.001).

The adjusted model with the total sample, which explained 24.2% of the variance in poor/fair self-perception of health, revealed that the factors most strongly associated with negative self-perception of health were being a woman (adjusted odds ratio (aOR): 1.65; 95% CI, 1.09–2.49), lack of familial support (aOR: 2.70; 95% CI, 1.23–5.89), BMI > 40 kg/m^2^ (aOR: 2.69; 95% CI, 1.22–5.94), mood disorders (aOR: 2.05; 95% CI, 1.26–3.34), and low physical activity (aOR: 2.23; 95% CI, 1.50–3.32) (Figure 1).

Table 2 shows the distribution of negative self-perceived health according to different subgroups and the crude OR of each subgroup stratified by sex. Table 3 presents the adjusted analysis of factors associated with negative self-perceived health by sex.

### 3.1. Men

The adjusted model for men had a Nagelkerke R^2^ of 24.3%. The factors most strongly associated with negative self-perception of health in men (Table 3) were age < 60 years (aOR: 4.30; 95% CI, 1.31–14.11), nonfamilial support (aOR: 7.96; 95% CI, 2.10–30.11), past treatment with ACEIs/ARBs (aOR: 3.24; 95% CI: 1.05–10.04), having a mood disorder (aOR: 2.34; 95%: 1.13–6.64), and low physical activity (aOR: 3.34; 95% CI: 1.80–6.20).

### 3.2. Women

The adjusted model for women had a Nagelkerke R^2^ of 24.1%. Women with cancer (OR: 5.05; 95% CI, 1.51–16.86), a BMI over 40 kg/m^2^ (OR: 4.10; 95% CI, 1.50–11.18), and those currently receiving antiplatelet therapy (OR: 3.16; 95% CI, 1.16–8.64) were more likely to have a negative self-perception of health. Table 3 shows the selected adjusted variables. The complete models are reflected in Supplementary Table S4.

Both models were adjusted for alcohol, microalbuminuria, retinopathy, neuropathy, respiratory disease, DPP-4 inhibitors, SGLT2 inhibitors, insulin, antihypertensive therapies, non-ACEIs/ARBs, psychiatric medications, analgesics, education level, atrial fibrillation, corticosteroids, immunomodulators, chronic kidney disease, cardiovascular disease, psychotic disorders, mood disorders, mood disorder medications, anticancer medications, sulfonylureas, and polypharmacy.

**Figure 2:**
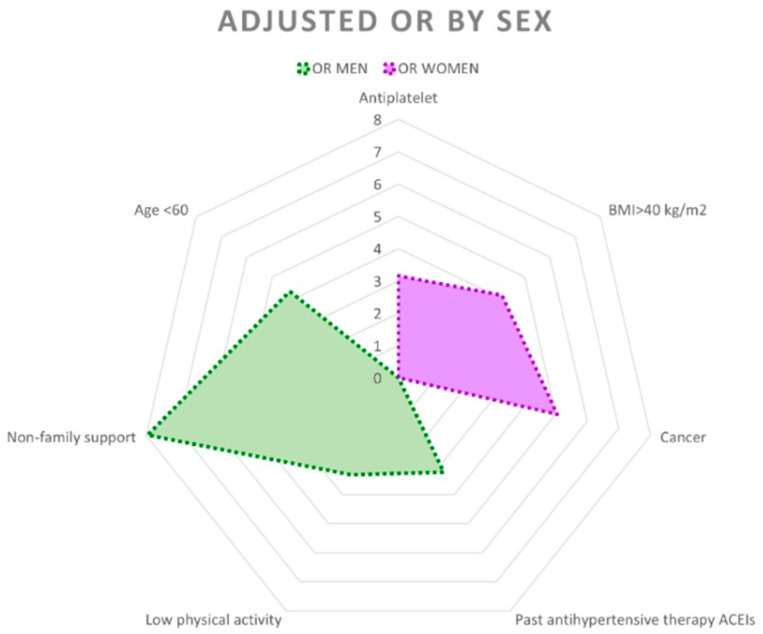
Radial graph showing adjusted odds ratios (ORs) with *p* < 0.05 and 95% confidence intervals by sex.

Figure 2 shows the differences in the factors that increase the probability of perceiving poor health between the sexes.

## 4. Discussion

This study revealed that approximately 26% of patients had negative self-perceived health, with women’s perceptions of their health being worse.

The overall data indicated that the factors associated with a poorer perception of health were low physical activity, mood disorders (anxiety or depression), being morbidly obese, having nonfamilial support, and being female. However, it is important to note that these factors affect men and women differently.

To the best of our knowledge, this study represents the first attempt to investigate the factors associated with self-perceived health in individuals with newly diagnosed diabetes mellitus.

Physical inactivity is a well-established risk factor that exacerbates both the clinical progression of diabetes and patient-reported health status. Our results reaffirm previous studies, which have demonstrated that insufficient physical activity is strongly correlated with poorer self-rated health [25,26,27,28]. Several reviews consistently emphasize the role of exercise in improving physical health and quality of life in diabetes management and other chronic diseases [29]. Exercise has been shown to have efficacy in reducing postprandial glucose excursions within just a few days. Exercise and physical activity should be recommended for all individuals with diabetes as part of their overall management of glycemic control and health [30,31].

The significant association between mood disorders and perceived poor/fair health underscores the crucial role that mental health plays in diabetes care. Depression and anxiety are prevalent comorbidities in individuals with diabetes and have been shown to negatively affect glycemic control, self-management, and overall health perception [32]. Our findings align with several epidemiological studies and Spanish studies that highlight mood disorders associated with poor/fair perceived health [33].

Obesity, particularly morbid obesity (BMI ≥ 40), is a significant cause of adverse health perceptions [34]. Severe obesity compounds diabetes-related complications and limits physical functioning, worsening patient-reported health [35]. Our findings are consistent with several studies that demonstrated a clear association between BMI and poorer health perception in patients with diabetes [36]. In Spain, where the prevalence of obesity is high, public health initiatives targeting weight management are critical components of comprehensive diabetes care [37].

A lack of familial support as a predictor of negative self-perception of health emphasizes the importance of social determinants in chronic disease management. Social isolation and lack of close support networks impair self-care behaviors and increase psychological distress in patients with diabetes [38]. Studies in our Mediterranean context, with solid family networks, even corroborate the impact of social support systems on patient adherence, lifestyle changes, and mental well-being, offering opportunities for family-based and community interventions to improve outcomes [39].

The association between female sex and negative self-perception of health in individuals with newly diagnosed diabetes is consistent with the findings of previous studies in populations with diabetes [40] and the general population [41,42]. An explanation for this phenomenon is that women commonly have multiple roles, including caregiving, household chores, and professional duties, often without adequate support. This role could generate chronic stress and deterioration in both mental and physical health, contributing to poorer self-perceived health than that of men.

Regarding sex differences in women, morbid obesity, cancer, and antiplatelet treatment were most strongly associated with a worse perception of health. However, in men, having nonfamilial support, being under 60 years of age, having low physical activity, having mood disorders, and receiving previous treatment with ACEI/ARB drugs were the factors associated with poorer health perceptions.

The smaller sample size in the female subgroup compared to the male subgroup may have influenced the observed sex differences with statistical significance in males but not in females.

Low to moderate adherence to a Mediterranean diet was not associated with poorer self-perceived health in the overall sample or in either sex subgroup, contrary to other previous studies [43], although there are contradictory results [13]. Therefore, our hypothesis of a positive association between high adherence to a Mediterranean diet and better self-perceived health was not supported.

Previous studies have shown that women tend to have a poorer perception of their health than men, and this trend has also been observed in the general Spanish population and in people with diabetes. According to data from the National Statistics Institute (INE), in 2022 32.3% of women stated that their health was fair, bad, or very bad. In contrast, the percentage of men who rated their health as very good or good was higher than that of women [44].

A study carried out in Catalonia among people aged 60 or older revealed that 57.3% of women reported poor health, whereas 43.6% of men reported poor health. Women suffer more disabilities (41.2% compared with 28.7% of men) and more chronic diseases (92.2% compared with 85.6% of men) [10]. These factors were not the ones most strongly associated with poor health perceptions among the women in our study.

According to the INE, single women over 65 years of age have a poorer perception of health, and our study also showed that situations of poor social and family support affect this index.

In previous studies on patients with diabetes, women reported lower quality of life than men did. In addition, high socioeconomic status, having social support, and having a partner were associated with better quality of life scores, which is consistent with our study [45].

Although the literature shows a relationship between a lack of social/family support and a low perception of health, in our study this association was found specifically in men but not in women.

Another factor associated with poor self-perception of health in previous studies was morbid obesity. Our work supported this finding, specifically in women. Obesity leads to comorbidities, deterioration of self-image and self-esteem, and loss of ability, and women with diabetes and obesity experience higher rates of depression, anxiety, and eating disorders than men do [46]. Furthermore, obesity is associated with insulin resistance, and the quality of life of patients with insulin resistance is worse than that of patients without it [47].

In relation to cancer, the disease itself, the side effects of drugs, and the associated mood disorders reduce quality of life [48]. In our study, this association was found only in women.

Our last variable associated with poor self-perception of health in women was antiplatelet treatment, which could be explained by the presence of cardiovascular disease and other associated factors rather than by the treatment itself.

Among the factors associated with poor health perceptions in men, a lack of family support stands out. A national study on the effect of social support on mortality in older people reported greater mortality in men who lived with relatives who were not their partner [49]. Another Brazilian study revealed that family support improved men’s perceptions of health [50].

In our study, men under 60 years of age with low physical activity had a low perception of health. Previous analyses in the same sample revealed lower physical activity and greater morbid obesity in this age group, which could explain these findings.

Another variable associated with poor self-perceived health in men was previous treatment with ACEIs/ARBs. This association may be attributed to the discontinuation of these medications due to severe hypotension, end-stage renal disease, or advanced-stage disease, although the size of this subgroup of patients was very small, and conclusions were difficult to draw.

Treatment with insulin was not related to a worse perception of health in our study, contrary to what is reflected in the literature [17], perhaps due to the low percentage of patients with this drug (11.6%), the greater comfort and safety of the new insulins, and the short duration of treatment.

With respect to diabetes-related comorbidities, previous studies have shown that quality of life is worse in patients with retinopathy, neuropathy, nephropathy, foot ulcers, and multiple complications of DM [18]. However, in our study, there was no high frequency of retinopathy or neuropathy, and these factors were not associated with poor self-perception of health.

Surprisingly, mood disorders such as anxiety and depression were associated with poor global health perceptions both overall and in men but not in women. This discrepancy is likely due to the smaller number of women included in the study than men, as previously mentioned; if the sample sizes were similar, these associations would likely have reached statistical significance in women as well.

There are sex differences in many diseases, including prevalence, clinical presentation, predisposing factors, and response to treatment [51]. There are also differences in the perceptions of health and related factors, as demonstrated in this study and many others. Men and women are different and should be considered as such in research studies to achieve the best results in personalized medicine.

Other factors, such as polypharmacy, antipsychotic use, psychosis, and corticosteroid use, were not associated with poor or fair self-perceived health in this study. Previous research using national surveys [52,53] and cross-sectional samples [54] has reported an association between polypharmacy and poorer self-perceived health. The lack of a significant association in our study may be due to differences in sample characteristics, particularly the higher proportion of males and older participants, as demographic factors like age and sex influence health perceptions and outcomes.

A transnational analysis from the World Health Surveys of Mental Health found that a history of psychotic experiences was significantly linked to higher odds of poor self-perceived mental health (OR = 1.5; 95% CI: 1.2–1.9) and poor physical health (OR = 1.3; 95% CI: 1.0–1.7), even after controlling for general medical or psychiatric conditions [55]. However, our study did not observe these associations, likely due to the small number of psychosis cases in our sample.

Corticosteroid use showed an initial crude association with poor self-perceived health, but this was not statistically significant after adjusting for comorbidities, sex, and age. Although studies in rheumatic disease populations have reported such associations [56], no research has been conducted in healthy or diabetic populations to our knowledge.

This study has several limitations. Many clinical researchers have participated in recruitment, and all patients with newly diagnosed diabetes were selected. However, not all of them could be included, which may introduce selection bias. In addition, the questionnaires were not self-administered, so participants’ responses may not have been entirely honest.

Another limitation of this study is its cross-sectional nature, which prevents the establishment of causal relationships between associated factors and self-perception of health, with a consequent reduction in impact on clinical decision-making or the formulation of social and health policies.

Finally, the logistic regression model explained 24% of the variability in poor/fair self-perception of health. This finding indicates that the variables included in the model explain nearly one-quarter of the differences observed in health perceptions among the studied population; if additional variables, such as socioeconomic status, housing conditions, employment circumstances, and family caregiving responsibilities, had been incorporated into both the overall and sex-stratified models, the explained variance would likely have increased. Nevertheless, the model’s explanatory power, accounting for 24% of the variability in self-perceived health, is modest, yet consistent with its multifactorial nature. Notably, approximately 76% of the variance remains unexplained, a finding that aligns with previous research in this field. This fact underscores the complex and multidimensional interplay of biological, psychological, and social factors that shape individuals’ perceptions of their health.

The study also has several strengths: the large sample size allows stratification by sex and increased statistical power, there are few publications in the studied population of patients with early-onset diabetes, and, finally, the representativeness of the sample, due to its heterogeneity and the participation of 12 health centers with different socioeconomic levels.

Understanding the factors associated with poor/fair health is a key objective for healthcare systems. Improving this perception is associated with better quality of life and has been shown to reduce morbidity and mortality. Studies such as the one we present can help identify areas for improvement in health education and policies that promote healthy lifestyles, accessible physical activity, affordable prices for a Mediterranean diet, and work–life balance, implement gender policies, and improve educational and social levels.

Addressing the differences between men and women is important. For men, the promotion of regular physical activity and access to mental health programs, as well as nurturing social and family connections, can have a profoundly positive impact. For women, focusing on obesity prevention and providing comprehensive support to those diagnosed with cancer are key strategies to promote a positive self-perception of health.

Finally, more studies are necessary to reinforce these results and analyse whether the measures adopted have positive consequences. Furthermore, this study could be extrapolated to different geographic populations with different lifestyles and to other subgroups of patients with diabetes. Future studies could also focus on gender differences and the biological factors that explain these differences.

## 5. Conclusions

In patients newly diagnosed with diabetes mellitus, the prevalence of poor/fair self-perceived health is comparable to that observed in the general population. Sex-specific patterns were identified: cancer and morbid obesity were more strongly associated with poor/fair health perception in women, whereas lack of family support, mood disorders, younger age, and low physical activity were more prominent in men. Due to the cross-sectional nature of the study, causal inferences cannot be drawn. The model explained 24% of the variability in self-perceived health, due to its multifactorial complexity. These findings highlight the need for tailored interventions addressing psychosocial and lifestyle factors in early diabetes care. Moreover, further research is necessary to understand the determinants of perceived health in this population fully.

## Figures and Tables

**Table 1:** Proportion of negative self-perceived health (fair/poor) and a crude analysis of its associated factors.

	Negative Self-Perceived Health (%)	*p*-Value	Crude OR (95% CI)	*p*-Value
**Sex**				
Men (*n* = 455)	20.2	<0.001	1	
Women (*n* = 341)	33.4		1.98 (1.44–2.73)	<0.001
**Age Group**				
≤60 (*n* = 378)	26.2	0.373	1	
61–75 (*n* = 303)	23.8		0.88 (0.62–1.25)	0.465
>75 (*n* = 115)	30.4		1.23 (0.78–1.95)	0.371
**Education**				
Superior education (*n* = 398)	21.9		1	
Elementary education (*n* = 398)	29.9	0.010	1.53 (1.11–2.10)	0.010
**Social Support**				
Family (*n* = 721)	24.0		1	
None (*n* = 39)	43.6	0.001	2.45 (1.27–4.72)	0.001
Yes, but not family social support (*n* = 36)	44.4		2.53 (1.29–5.00)	0.001
**BMI Categories**				
<25 kg/m^2^ (*n* = 120)	27.5	<0.001	1	
25–29 kg/m^2^ (*n* = 272)	21.7		0.73 (0.45–1.20)	0.212
30–34 kg/m^2^ (*n* = 248)	23.8		0.82 (0.50–1.35)	0.441
35–40 kg/m^2^ (*n* = 102)	26.5		0.95 (0.52–1.72)	0.863
>40 kg/m^2^ (*n* = 54)	51.9		2.84 (1.46–5.53)	0.002
**CKD**				
No (*n* = 717)	24.0		1	
Yes (*n* = 79)	43.0	<0.001	2.39 (1.49–3.86)	<0.001
**Retinopathy**				
No (*n* = 722)	24.7		1	
Yes (*n* = 74)	37.8	0.021	1.86 (1.13–3.07)	0.015
**Neuropathy**				
Yes (*n* = 57)	42.1	<0.001	2.23 (1.28–3.87)	<0.01
No (*n* = 739)	24.6		1	
**CVD**				
No (*n* = 639)	22.1		1	
Yes (*n* = 157)	41.4	<0.001	2.50 (1.73–3.61)	<0.001
**Hypertension**				
No (*n* = 385)	22.3		1	
Yes (*n* = 411)	29.2	0.026	1.43 (1.04–1.98)	0.028
**Atrial Fibrillation**				
No (*n* = 723)	18.4		1	
Yes (*n* = 73)	35.4	0.001	1.91 (1.15–3.15)	0.012
**Respiratory Diseases**				
No (*n* = 658)	23.7		1	
Yes (*n* = 138)	36.2	<0.01	1.83 (1.24–2.70)	<0.01
**Psychotic Disorders**				
No (*n* = 756)	24.6		1	
Yes (*n* = 40)	50.0	0.001	3.07 (1.61–5.82)	0.001
**Mood Disorders**				
No (*n* = 578)	20.4		1	
Yes (*n* = 218)	40.4	<0.001	2.64 (1.89–3.70)	<0.001
**Cancer**				
No (*n* = 708)	24.4		1	
Yes (*n* = 88)	37.5	0.012	1.86 (1.17–2.95)	<0.01
**DPP-4 Inhibitors**				
Never (*n* = 612)	23.9	0.036	1	
Yes, but not currently (*n* = 68)	36.8		1.86 (1.10–3.14)	0.021
Yes, currently (*n* = 116)	30.2		1.38 (0.89–2.14)	0.150
**SGLT2 Inhibitors**				
Never (*n* = 568)	24.6	0.013	1	
Yes, but not currently (*n* = 42)	45.2		2.53 (1.34–4.78)	<0.01
Yes, currently (*n* = 186)	25.3		1.03 (0.71–1.51)	0.865
**Antihypertensives Therapies, ACEIs/ARBs**				
Never (*n* = 355)	37.4	<0.001	1	
Yes, but not currently (*n* = 55)	49.1		3.48 (1.94–6.25)	<0.001
Yes, currently (*n* = 386)	26.4		1.30 (0.93–1.82)	0.133
**Antihypertensive Therapies, Non-ACEIs/ARBs**				
Never (*n* = 556)	22.7	<0.01	1	
Yes, but not currently (*n* = 82)	39.0		2.18 (1.34–3.55)	<0.01
Yes, currently (*n* = 158)	30.4		1.49 (1.01–2.01)	0.047
**Antiplatelet Drugs**				
Never (*n* = 613)	22.2	<0.001	1	
Yes, but not currently (*n* = 54)	38.9		2.23 (1.25–3.98)	<0.01
Yes, currently (*n* = 129)	38.0		2.15 (1.44–3.22)	<0.001
**Psychiatric Drugs**				
Never (*n* = 730)	24.4	<0.01	1	
Yes, but not currently (*n* = 34)	38.2		1.92 (0.94–3.91)	0.073
Yes, currently (*n* = 32)	46.9		2.74 (1.34–5.59)	<0.01
**Mood Disorder Drugs**				
Never (*n* = 638)	23.4	<0.001	1	
Yes, but not currently (*n* = 52)	25.0		1.09 (0.57–2.10)	0.788
Yes, currently (*n* = 106)	41.5		2.33 (1.52–3.57)	<0.001
**Corticosteroids**				
Never (*n* = 553)	23.9	0.041	1	
Yes, but not currently (*n* = 180)	27.8		1.23 (0.84–1.79)	0.292
Yes, currently (*n* = 63)	38.1		1.96 (1.14–3.38)	0.015
**Anticancer Medication**				
Never (*n* = 697)	24.1	0.001	1	
Yes, but not currently (*n* = 31)	22.6		0.92 (0.39–2.17)	0.846
Yes, currently (*n* = 68)	45.6		2.64 (1.59–4.38)	<0.001
**Immunomodulators**				
Never (*n* = 711)	23.9	0.001	1	
Yes, but not currently (*n* = 27)	40.7		2.19 (0.99–4.81)	0.051
Yes, currently (*n* = 58)	43.1		2.41 (1.39–4.17)	<0.01
**Glinides**				
Never (*n* = 742)	25.2	0.068	1	
Yes, but not currently (*n* = 12)	16.7		0.59 (0.13–2.73)	0.503
Yes, currently (*n* = 42)	40.5		2.02 (1.07–3.82)	0.031
**Sulfonylureas**				
Never (*n*= 699)	23.7	<0.001	1	
Yes, but not currently (*n* = 65)	47.7		2.93 (1.75–4.91)	<0.001
Yes, currently (*n* = 32)	28.1		1.26 (0.57–2.77)	0.571
**Polypharmacy**				
No (*n* = 435)	17.9	<0.001	1	
Yes (*n* = 361)	35.5		2.51 (1.81–3.49)	<0.001
**Mediterranean Diet**				
High adherence, ≥11 (*n* = 175)	20.6	0.126	1	
Medium adherence, 6–10 (*n* = 559)	26.8		1.42 (0.94–2.14)	0.098
Low adherence, 0–5 (*n* = 62)	32.3		1.84 (0.96–3.51)	0.065
**Physical Activity**				
Moderate or high (*n* = 399)	18.8	<0.001	1	
Low (*n* = 397)	33.0		2.13 (1.53–2.95)	<0.001
**FPG**				
≥100 mg/dL	25.9	0.920	1	
<100 mg/dL	25.0		0.95 (0.37–2.44)	0.920
**HbA1c**				
≥7%	24.0	0.160	1	
<7%	28.4		1.26 (0.91–1.73)	0.160
**LDL Cholesterol**				
≥100 mg/dL	27.9	0.071	1	
<100 mg/dL	22.0		0.83 (0.61–1.15)	0.071

%: proportion of people with poor/fair self-reported health status by category and subcategory. CKD: chronic kidney disease. CVD: cardiovascular disease. DPP-4: dipeptidyl peptidase-4. SGLT2: sodium–glucose cotransporter type 2. FPG: fasting plasma glucose. 95% CI: 95% confidence interval.

**Table 2:** Proportion of negative self-perception and a crude analysis of the associated factors by sex.

	Men (*n* = 455)				Women (*n* = 341)			
	Negative Self-Perception (%)	*p*-Value	Crude OR (95% CI)	*p*-Value	Negative Self-Perception (%)	*p*-Value	Crude OR (95% CI)	*p*-Value
**Age Group**		0.042				0.200		
>75 years	12.2		1		40.5		1	
61–75 years	16.2		1.39 (0.50–3.85)	0.524	34.7		0.78 (0.43–1.41)	0.408
<60 years	24.7		2.36 (0.88–630)	0.086	28.7		0.59 (0.33–1.06)	0.079
**Education**		0.379				0.034		
Superior education	18.7		1		27.2		1	0.035
Elementary education	22.1		1.23 (0.78–1.94)	0.379	38.1		1.65 (1.04–2.63)	
**Social Support**		<0.001				0.094		
Familial	18.3		1		31.9		1	
None	29.4		1.86 (0.64–5.42)	0.258	54.5		2.56 (1.07–6.14)	0.035
Yes, but not familial	55.6		5.57 (2.13–14.57)	<0.001	33.3		1.07 (0.39–2.93)	0.899
**BMI Categories**		0.026				0.019		
<25 kg/m^2^	26.9		1		27.9		1	
25–29 kg/m^2^	17.7		0.58 (0.28–1.21)	0.149	27.8		0.99 (0.50–1.95)	0.981
30–34 kg/m^2^	16.0		0.52 (0.25–1.09)	0.084	37.0		1.51 (0.77–2.98)	0.232
35–40 kg/m^2^	24.2		0.87 (0.37–2.02)	0.739	30.0		1.11 (0.47–2.61)	0.819
>40 kg/m^2^	42.9		2.04 (0.71–5.87)	0.188	57.6		3.50 (1.47–8.36)	<0.001
**Alcohol**		0.011				0.777		
Teetotal	8.9		1		33.9		1	
Heavy drinker	28.1		2.07 (1.19–3.58)	0.010	40.0		1.30 (0.45–3.78)	0.633
Drinker, but not risky	23.4		2.64 (1.26–5.52)	0.010	31.4		0.89 (0.54–1.47)	0.653
**Microalbuminuria**		<0.001				0.345		
No	17.4		1		34.2		1	
Yes	36.9		2.77 (1.57–4.89)	<0.001	25.8		0.67 (0.29–1.55)	0.348
**CKD**		0.009				0.014		
No	18.6		1		31.6		1	
Yes	35.7		2.42 (1.23–4.78)	0.010	51.4		2.32 (1.17–4.62)	0.017
**Retinopathy**		0.006				0.318		
No	18.4		1		32.7		1	
Yes	35.4		2.13 (1.28–4.62)	0.007	42.3		1.51 (0.67–3.40)	0.321
**Neuropathy**		0.003				0.245		
No	18.7		1		32.6		1	
Yes	40.6		2.98 (1.41–6.29)	0.004	44.0		1.63 (0.71–3.70)	0.248
**CVD**		<0.001				0.002		
No	15.3		1		30.1		1	
Yes	36.1		3.14 (1.92–5.12)	<0.001	53.1		2.62 (1.41–4.84)	<0.01
**Atrial Fibrillation**		0.269				0.013		
No	19.6		1		31.4		1	
Yes	26.8		1.51 (0.73–3.14)	0.272	53.1		2.48 (1.19–5.16)	0.016
**Respiratory Diseases**		0.026				0.052		
No	18.4		1		31.0		1	
Yes	29.7		1.88 (1.07–3.30)	0.028	43.8		1.73 (0.99–3.01)	0.054
**Psychotic Disorders**		0.010				0.051		
No	19.3		1		32.0		1	
Yes	46.7		3.65 (1.29–10.36)	0.015	52.0		2.31 (1.02–5.23)	0.046
**Mood Disorders**		<0.001				0.007		
No	16.5		1		27.7		1	
Yes	39.5		3.10 (1.83–5.27)	<0.001	41.7		1.87 (1.18–2.95)	<0.01
**Cancer**		0.078				0.009		
No	189		1		31.4		1	31.4
Yes	28.8		1.73 (0.94–3.21)	0.081	55.2		2.69 (1.25–5.80)	55.2
**DPP-4 Inhibitors**		0.032				0.135		
Never	18.5		1		30.8		1	
Yes, but not currently	42.9		2.45 (1.23–4.87)	0.011	38.5		1.40 (0.61–3.22)	0.426
Yes, currently	19.4		1.06 (0.55–2.06)	0.862	44.9		1.83 (0.98–3.40)	0.057
**SGLT2 Inhibitors**		0.001				0.738		
Never	18.5		1		32.3		1	
Yes, but not currently	48.3		4.12 (1.88–9.01)	<0.001	38.5		1.31 (0.42–4.12)	0.644
Yes, currently	17.9		0.96 (0.55–1.68)	0.885	36.5		1.21 (0.70–2.07)	0.500
**Insulin**		0.014				0.899		
Never	17.7		1		33.6		1	
Yes, but not currently	23.5		1.43 (0.62–3.31)	0.400	35.7		1.10 (0.49–2.48)	0.819
Yes, currently	33.9		2.39 (1.31–4.36)	0.005	30.0		0.86 (0.39–1.88)	0.706
**Antihypertensive Therapies, ACEIs/ARBs**		<0.001				0.062		
Never	16.5		1		28.9		1	
Yes, but not currently	45.9		4.30 (2.04–9.05)	<0.001	55.6		3.08 (1.14–8.33)	0.027
Yes, currently	19.3		1.21 (0.74–2.00)	0.451	35.1		1.33 (0.83–2.13)	0.235
**Antihypertensive Therapies, Non-ACEIs/ARBs**		0.007				0.080		
Never	17.0		1	0.002	29.9		1	
Yes, but not currently	6.3		2.65 (1.10–4.99)	0.238	46.4		2.03 (0.92–4.48)	0.080
Yes, currently	22.5		1.42 (0.79–2.52)		40.6		1.60 (0.92–2.78)	0.096
**Antiplatelet Drugs**		0.001				0.004		
Never	16.0		1		29.5		1	
Yes, but not currently	29.0		2.15 (0.94–4.94)	0.070	52.2		2.60 (1.10–6.13)	0.029
Yes, currently	32.6		2.55 (1.51–4.31)	<0.001	51.4		2.52 (1.26–5.04)	0.009
**Psychiatric Drugs**		<0.001				0.508		
Never	18.0		1		32.7		1	
Yes, but not currently	34.6		2.42 (1.04–5.64)	0.041	50.0		2.06 (0.56–8.39)	0.314
Yes, currently	52.9		5.14 (1.92–13.76)	0.001	40.0		1.37 (0.48–3.96)	0.559
**Mood Disorder Drugs**		0.044				0.092		
Never	19.0		1		30.3		1	
Yes, but not currently	16.0		0.81 (0.27–2.43)	0.707	33.3		1.15 (0.49–2.68)	0.748
Yes, currently	36.1		2.40 (1.16–4.96)	0.018	44.3		1.83 (1.06–3.15)	0.030
**Analgesics**		0.014				0.102		
Never	26.2		1		30.0		1	
Yes, but not currently	15.4		0.52 (0.27–0.99)	0.045	26.8		0.86 (0.36–2.06)	0.727
Yes, currently	26.7		1.02 (0.52–2.02)	933	38.3		1.45 (0.63–3.34)	0.384
**Corticosteroids**		0.758				0.031		
Never	19.8		1		30.2		1	
Yes, but not currently	19.8		1.00 (0.54–1.83)	0.989	34.3		1.21 (0.73–2.00)	0.467
Yes, currently	35.0		1.35 (0.61–3.00)	0.464	55.6		2.89 (1.28–6.50)	0.011
**Anticancer Medication**		0.003				0.099		
Never	18.7		1		31.4		1	
Yes, but not currently	11.1		0.54 (0.12–2.41)	0.423	38.5		1.36 (0.42–4.28)	0.595
Yes, currently	85.3		3.11 (1.53–6.31)	0.002	50.0		2.18 (1.05–4.55)	0.037
**Immunomodulators**		0.001				0.176		
Never	17.7		1		32.0		1	
Yes, but not currently	42.9		3.50 (1.42–8.61)	0.006	33.3		1.06 (0.19–5.89)	0.946
Yes, currently	37.5		2.80 (1.31–5.98)	0.008	50.0		2.12 (0.95–4.75)	0.067
**Sulfonylureas**		<0.01				0.024		
Never	18.0		1		31.4		1	
Yes, but not currently	41.0		3.17 (1.59–6.30)	0.001	57.7		2.97 (1.32–6.72)	<0.01
Yes, currently	25.0		1.52 (0.48–4.84)	0.480	31.3		0.99 (0.34–2.93)	0.987
**Polypharmacy**		<0.001				<0.001		
Yes	13.8		1		43.4		2.43 (1.53–3.86)	<0.001
No	28.7		2.51 (1.57–4.01)	<0.001	24.0		1	
**Mediterranean Diet**		0.087				0.095		
High adherence, ≥11	18.4		1		23.4		1	
Medium adherence, 6–10	19.0		1.04 (0.58–1.87)	0.894	36.7		1.90 (1.06–3.42)	0.032
Low adherence, 0–5	32.6		2.15 (0.97–4.79)	0.061	31.3		1.49 (0.46–4.86)	0.508
**Physical Activity**		0.001				0.008		
Moderate or high	14.5		1		25.8		1	
Low	27.1		2.18 (1.37–3.49)	0.001	39.5		1.87 (1.18–2.99)	<0.01

%: proportion of poor/fair self-reported health among each category and subcategory. BMI: body mass index. CKD: chronic kidney disease. CVD: cardiovascular disease. DPP-4 inhibitors: dipeptidyl peptidase-4 inhibitors. SGLT2 inhibitors: sodium–glucose cotransporter type 2 inhibitors. Crude OR: crude odds ratio. 95% CI: 95% confidence interval.

**Table 3:** Adjusted analysis of the factors associated with negative self-perception by sex.

	Men (*n* = 445)		Women (*n* = 341)	
	Adjusted OR (95% CI)	*p*-Value	Adjusted OR (95% CI)	*p*-Value
**Age Group**				
>75 years	1		1	
61–75 years	2.22 (0.67–7.33)	0.189	0.78 (0.39–1.59)	0.493
<60 years	**4.30 (1.31–14.11)**	0.016	0.75 (0.35–1.60)	0.456
**Social Support**				
Familial	1		1	
Yes, but no familial	**7.96 (2.10–30.11)**	0.002	1.43 (0.46–4.46)	0.542
None	1.15 (0.30–4.44)	0.843	1.98 (0.70–5.60)	0.196
**BMI Categories**				
<25 kg/m^2^	1		1	
25–29 kg/m^2^	0.61 (0.23–1.64)	0.325	0.82 (0.37–1.84)	0.636
30–34 kg/m^2^	0.43 (0.16–1.18)	0.102	1.37 (0.60–3.12)	0.451
35–40 kg/m^2^	0.71 (0.24–2.12)	0.538	1.15 (0.42–3.14)	0.780
>40 kg/m^2^	1.35 (0.36–5.02)	0.654	**4.10 (1.50–11.18)**	0.006
**Cancer**				
No	1		1	
Yes	0.64 (0.20–2.05)	0.452	**5.05 (1.51–16.86)**	0.009
**Mood Disorders**				
No	1		1	
Yes	**2.73 (1.13–6.64)**	0.026	1.81 (0.94–3.50)	0.077
**Antihypertensive Therapies, ACEIs/ARBs**				
Never	1		1	
Yes, but not currently	**3.24 (1.05–10.04)**	0.041	0.66 (0.17–2.55)	0.551
Yes, currently	0.96 (0.47–1.96)	0.779	0.85 (0.44–1.65)	0.635
**Antiplatelet Drugs**				
Never	1		1	
Yes, but not currently	1.11 (0.28–4.42)	0.879	2.06 (0.61–6.98)	0.246
Yes, currently	1.13 (0.39–3.25)	0.818	**3.16 (1.16–8.64)**	0.025
**Physical Activity**				
Moderate or high	1		1	
Low	**3.34 (1.80–6.20)**	<0.001	1.66 (0.94–2.92)	0.082
**Mediterranean Diet**				
High adherence, ≥11	1		1	
Medium adherence, 6–10	1.01 (0.49–2.09)	0.985	1.45 (0.74–2.85)	0.278
Low adherence, 0–5	1.93 (0.72–5.18)	0.192	0.72 (0.17–2.95)	0.645

BMI: body mass index. ACEIs: angiotensin-converting enzyme inhibitors. ARBs: angiotensin II receptor blockers. 95% CI: 95% confidence interval. Bold means *p* ≤ 0.05.

## Data Availability

There are restrictions on data availability for the LADA-PC dataset because of the signed consent agreements around data sharing, which only allow access to external researchers for research following the project’s purposes once it has been completed. The requestors wishing to access the LADA-PC data used in this study can request it to the LADA-PC Steering Committee: lada.ap.research@gmail.com.

## Supplementary Materials

The following supporting information can be downloaded at: https://www.mdpi.com/article/10.3390/jcm14196770/s1.

## Abbreviations

The following abbreviations are used in this manuscript:

ACEIs/ARBs	Angiotensin-converting enzyme inhibitors/angiotensin receptor blockers
ADA	American Diabetes Association
aOR	Adjusted odds ratio
BMI	Body mass index
CI	Confidence interval
CKD	Chronic kidney disease
DM	Diabetes mellitus
DPP-4 inhibitors	Dipeptidyl peptidase-4 inhibitors
EMRs	Electronic medical records
HbA1c	Glycosylated haemoglobin, haemoglobin A1C
INE	National Statistics Institute (Instituto Nacional de Estadística)
IPAQ	International Physical Activity Questionnaire
LDL	Low-density lipoprotein
MEDAS	Mediterranean Diet Adherence Screener
MS	Metabolic syndrome
SGLT2	Sodium–glucose cotransporter type 2
