# Supplementary material for: Self-Perceived Health Status of Patients with Newly Diagnosed Diabetes in Spain: Associated Factors and Sex Differences
Source: J Clin Med. 2025 Sep 25;14(19):6770. doi: 10.3390/jcm14196770 (PMC12524603; doi:10.3390/jcm14196770)
Supplement: Supplementary file 1 [file jcm-14-06770-s001.zip › jcm-3817772-supplementary.pdf]

Supplementary Material

Supplementary Table S1: Definition of variables:

Anthropometric parameters, such as weight, height, waist circumference, and body mass index (BMI), were measured via standard methods [21].

Self-perceived health: Self-perceived health was assessed by asking the following question: "How would you rate your health?" The response options were "excellent", "very good", "good", "fair", and "poor". For analysis purposes, responses were dichotomised as "positive self-perceived health" (excellent, very good and good) or "negative self-perceived health" (fair and poor), following previous research on this topic.

Sociofamily support during illness: Patients were asked, "Who takes care of you when you are ill and need help? The options were "I have no one to turn to", "Your partner", "Another relative who is not your partner", "Someone else without family ties (neighbors, friends)", "Someone who is paid", "Social services" or "Other situations". These items were grouped into categories of "non-social-family support", "yes, but not familiar", and "yes, familiar". For the purposes of this analysis, "yes, but not familiar" was considered to indicate nonfamily support or a lack of family support, given the similar meanings of both concepts.

Educational level: no or incomplete primary education, primary education, junior and senior high school, basic and advanced vocational training, and university studies. These categories were stratified into two groups: basic education (no or incomplete or primary education) and higher education (all other categories).

Tobacco use: active smoker (current smoker, either daily or occasionally), never smoker, or ex-smoker (not smoked for at least 1 year).

Number of tobacco pack-years: It is calculated by multiplying the number of cigarette packs smoked per day by the number of years the individual has smoked.

Alcohol consumption: An electronic booklet tool was used to calculate daily and weekly alcohol consumption, classifying patients as heavy drinkers (>40 g of alcohol/day for men, >24 g for women), drinkers but not at risk (>0 and <40 g of alcohol/day for men, >0 and <24 g for women), and nondrinkers. The alcohol consumption volume was measured as follows: small glass, 125 ml; medium glass, 200 ml; large glass, 250 ml; mixed drink glass, 50 ml; beer bottle, 200 ml; and wine bottle, 750 ml.

Physical activity: A reduced scale of the International Physical Activity Questionnaire (IPAQ) was used, with the following categories: low (not performing any physical activity or insufficient activity performed to meet IPAQ category 2 or 3), moderate (≥ three sessions/week of vigorous physical activity for at least 20 minutes/day; or ≥ five sessions/week of moderate physical activity and/or walking for at least 30 minutes/day; or ≥ five sessions/week of any combination of walking and/or moderate and/or vigorous physical activity) and high physical activity (≥ three sessions/week of vigorous physical activity for at least 60 minutes/day, or ≥ seven sessions/week of any combination of walking and/or moderate and/or vigorous physical activity).

Mediterranean diet: A modified version of the Mediterranean Diet Adherence Screener (MEDAS) questionnaire was used to analyse adherence to the Mediterranean diet. Scores of 11-14 indicate high adherence. The modification involved adjusting wine consumption from two drinks per week instead of seven, as in the original MEDAS [22], to improve screening for alcohol consumption, since the questionnaire categorised risk on the basis of the type of drink and grams/week, not just the number of alcohol units.

Cardiovascular disease (CVD): Defined as cerebrovascular disease, coronary heart disease, or peripheral arterial disease.

Metabolic syndrome (MS): Defined according to the National Cholesterol Education Program Adult Treatment Panel III as the presence of at least three of the following components: 1) abdominal circumference ≥ 102 cm in men and ≥88 cm in women; 2) triglycerides ≥ 150 mg/dl; 3) blood pressure ≥ 130/85 mm/Hg or established hypertension; 4) HDL cholesterol < 40 mg/dl in men and < 50 mg/dl in women; and 5) fasting plasma glucose 110 to 126 mg/dl (6.11–6.99 mmol/L) or DM diagnosis [23].

Other comorbidities: microalbuminuria, chronic kidney disease (CKD), diabetic retinopathy, diabetic neuropathy, hypertension, hypercholesterolemia, hypertriglyceridaemia, atrial fibrillation,

heart failure, respiratory diseases (chronic obstructive pulmonary disease and asthma), mood disorders (anxiety and depression), psychotic disorders and cancer.

Therapies for DM: metformin, sulfonylureas, glitazones, alpha-glucosidase inhibitors, dipeptidyl peptidase-4 inhibitors (DPP-4 inhibitors), GLP-1 receptor analogues, sodium–glucose cotransporter type 2 inhibitors (SGLT2 inhibitors), glinides, and insulin.

Other drugs: ACEI/ARB antihypertensives, non-ACEI/ARB antihypertensives, lipid-lowering drugs, mood disorder drugs (anxiolytics, antidepressants), antipsychotics, antiplatelets, oral anticoagulants, analgesics, corticosteroids, anticancer drugs, and immunomodulators.

Polypharmacy: Defined as the regular use of five or more medications per day, as reported in most studies [24].

Laboratory parameters: blood glucose at diagnosis; HbA1c at diagnosis and in the last six months; the albumin/creatinine ratio; ketonuria at some time during disease progression; and total cholesterol, HDL and LDL cholesterol, and triglyceride levels.

Supplementary Figure S1. Flow chart of observational cross-sectional study

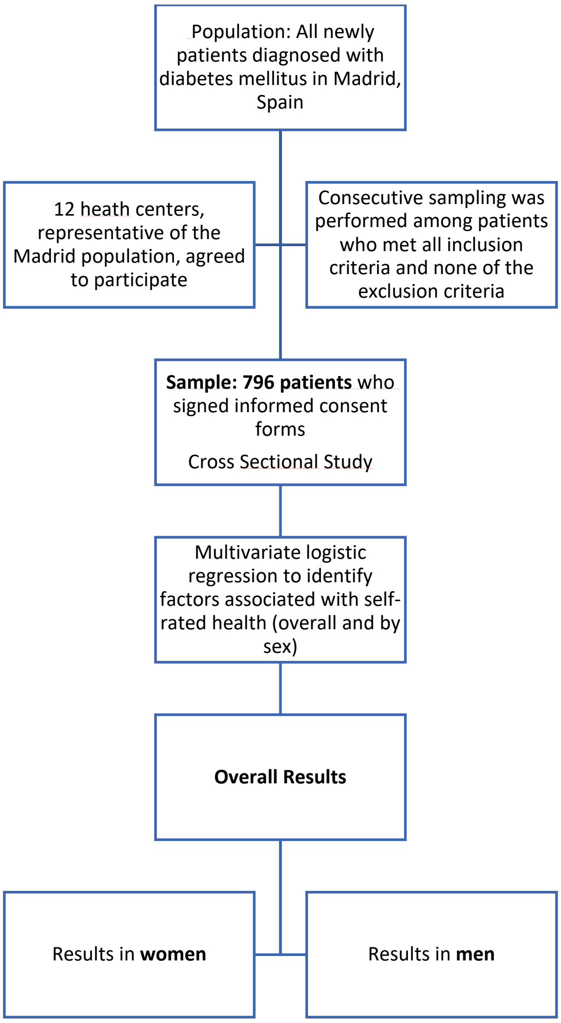

Supplementary Table S2. List of variables with absolute and relative frequency of missing values

	N	Number of missing	Total	% Missings
**Sex**	796	0	796	0,0
**Age**	796	0	796	0,0
**Alcohol (units per week)**	729	67	796	8,4
**Alcohol consumption**	796	0	796	0,0
**Tobacco use**	796	0	796	0,0
**Number tobacco packs per year**	425	371	796	46,6
**Education level**	796	0	796	0,0
**Social support**	796	0	796	0,0
**BMI categories**	796	0	796	0,0
**Chronic kidney disease**	796	0	796	0,0
**Retinopathy**	796	0	796	0,0
**Neuropathy**	796	0	796	0,0
**Cardiovascular disease**	796	0	796	0,0
**Hypertension**	796	0	796	0,0
**Atrial fibrillation**	796	0	796	0,0
**Respiratory diseases**	796	0	796	0,0
**Psychotic disorders**	796	0	796	0,0
**Mood disorders**	796	0	796	0,0
**Cancer**	796	0	796	0,0
**DPP-4 inhibitors**	796	0	796	0,0
**SGLT2-inhibitors**	796	0	796	0,0
**Antihypertensives ACEI/ARB**	796	0	796	0,0
**Antihypertensives non ACEI/ARB**	796	0	796	0,0
**Antiplatelet drugs**	796	0	796	0,0
**Psychiatric drugs**	796	0	796	0,0
**Mood disorder drugs**	796	0	796	0,0
**Corticosteroids**	796	0	796	0,0
**Anticancer medication**	796	0	796	0,0
**Immunomodulators**	796	0	796	0,0
**Glinides**	796	0	796	0,0
**Sulfonylureas**	796	0	796	0,0
**Metformin**	796	0	796	0,0
**Polipharmacy**	796	0	796	0,0
**Meditarranean diet**	796	0	796	0,0
**Physical activity**	796	0	796	0,0
**Fasting plasma glucose**	711	85	796	10,7
**HbA1c**	704	92	796	11,6
**Cholesterol total**	701	95	796	11,9
**HDL Cholesterol**	700	96	796	12,1
**LDL Cholesterol**	701	95	796	11,9
**Triglycerides**	700	96	796	12,1

Bold means significant the variables that have been included in the logistic regression model in the present study

Supplementary Figure S2. Missing values analysis

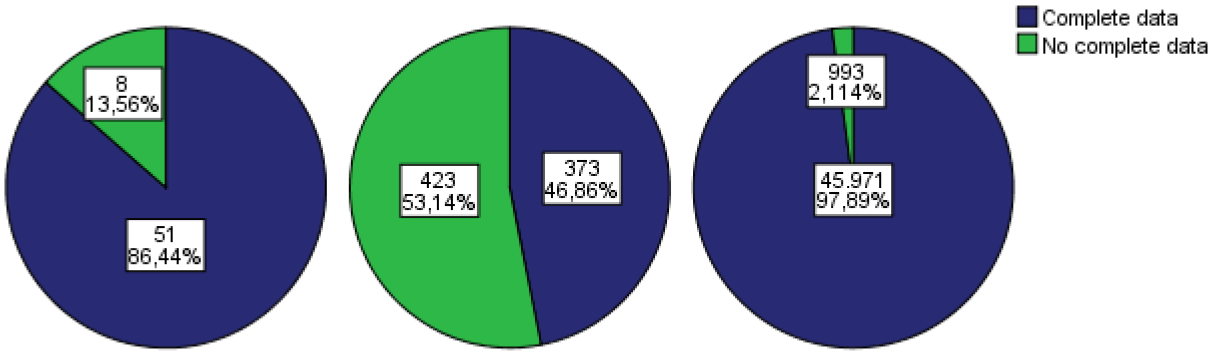

Variables **Cases** **Values**

Eight variables (13.56%) had missing values, 423 cases (53.14%) had missing values and 993 values from 45,971 (2.11%) were missing.

Supplementary Figure S3. Missing data pattern

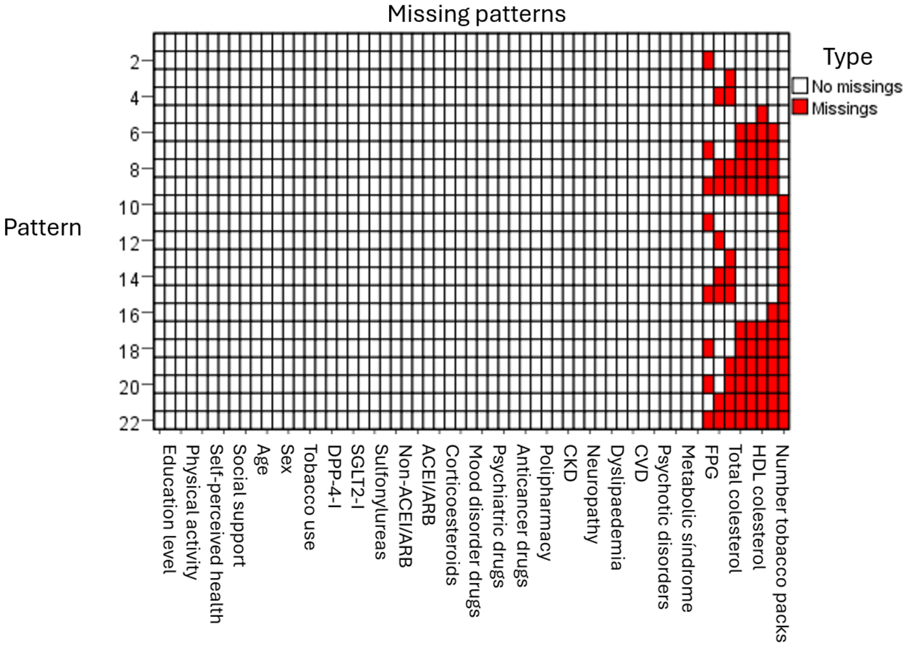

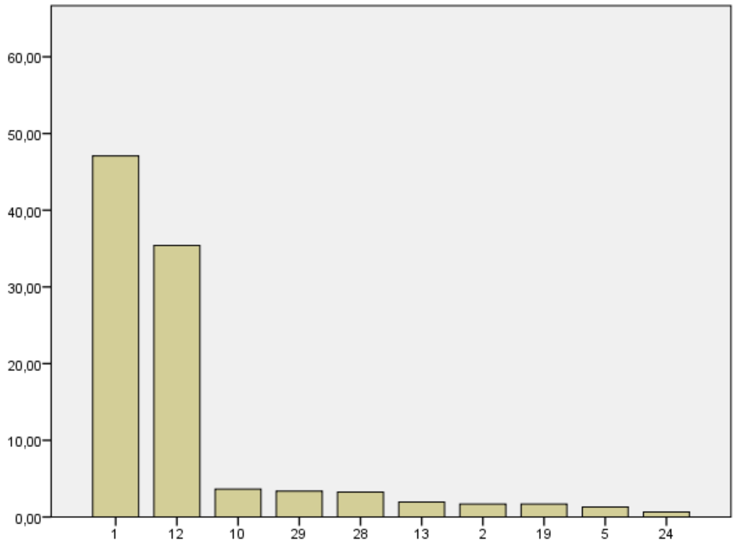

The most frequent pattern was 1 (no missing values), followed by pattern 2, associated with tobacco pack-years; all others occurred at low and similar frequencies, indicating that missing data were concentrated in a small number of variables. A non-monotone pattern was observed, and missing values were imputed using a single-pass, non-iterative mean substitution, as the affected variables were used solely for descriptive purposes. The mean was chosen over the median because the variables exhibited distributions close to normality, making the mean an appropriate and unbiased estimator. Variables FPG < 100 mg/dL, HbA1c < 7%, and LDL < 100 mg/dL were excluded from the multivariate logistic regression model due to a lack of significant association with poor/fair self-perceived health and limited clinical priority in newly diagnosed diabetes patients, who typically focus on understanding and implementing the lifestyle modifications required for disease management, thereby reducing overfitting. Tobacco pack-years (46.6% missing) was also excluded due to redundancy with tobacco use and to avoid multicollinearity and selection bias.

Supplementary Table S3. Distribution of quantitative variables stratified by self-perceived health status

	**Negative self-perceived health**	**Positive self-perceived health**	**p-value**
Age, *mean (SD)*	62.6 (11.0)	61.7 (11.0)	0.288
Age at diagnosis, *mean (SD)*	59.9 (11.0)	59.0 (11.0)	0.315
Pack-years of tobacco cigarette smoking, *mean (SD)*	25.6 (21.6)	24.8 (22.2)	0.764
Alcohol units per week, *mean (SD)*	3.3 (8.2)	3.8 (7.6)	0.496
BMI, *mean (SD)*	31.9 (7.0)	30.4 (5.4)	0.009
Fasting plasma glucose, *mean (SD)*	168.0 (86.9)	175.5 (83.0)	0.306
HbA1c (%), *mean (SD)*	7.6 (2.1)	7.8 (2.0)	0.164
Total cholesterol mg/dl, *mean (SD)*	183.4 (41.2)	172.2 (35.9)	0.002
LDL cholesterol mg/dl, *mean (SD)*	106.0 (37,8)	97.4 (32.4)	0.008
Triglycerides mg/dl, *mean (SD)*	153.1 (94.9)	139.7 (92.3)	0.099
Number of medications per day, *mean (SD)*	5.9 (3.2)	4.3 (2.6)	<0,001
MEDAS score, *mean (SD)*	2.8 (1.3)	2.6 (1.3)	0.201

Supplementary Table S4. Adjusted analysis of the factors associated with negative self-perception by sex

	**Men (n=455)**		**Women (n=341)**	
	**Adjusted OR (95% CI)**	**p value**	**Adjusted OR (95% CI)**	**p value**
**Age group**				
>75 years	1		1	
61-75 years	2.22 (0.67-7.33)	.189	0.78 (0.39-1.59)	.493
<60 years	**4.30 (1.31-14.11)**	.016	0.75 (0.35-1.60)	.456
**Social support**				
Familiar	1		1	
Yes, but no familiar	**7.96 (2.10-30.11)**	.002	1.43 (0.46-4.46)	.542
None	1.15 (0.30-4.44)	.843	1.98 (0.70-5.60)	.196
**BMI categories**				
<25 kg/m2	1		1	
25-29 kg/m2	0.61 (0.23-1.64)	0.325	0.82 (0.37-1.84)	.636
30-34 kg/m2	0.43 (0.16-1.18)	0.102	1.37 (0.60-3.12)	.451
35-40 kg/m2	0.71 (0.24-2.12)	0.538	1.15 (0.42-3.14)	.780
>40 kg/m2	1.35 (0.36-5.02)	0.654	**4.10 (1.50-11.18)**	.006
**Cancer**				
No	1		1	
Yes	0.64 (0.20-2.05)	0.452	**5.05 (1.51-16.86)**	.009
**Antihypertensives therapies**				
**ACEIs/ARBs**				
Never	1		1	
Yes, but not currently	**3.24 (1.05-10.04)**	.041	0.66 (0.17-2.55)	.551
Yes, currently	0.96 (0.47-1.96)	.779	0.85 (0.44-1.65)	.635
**Antiplatelet drugs**				
Never	1		1	
Yes, but not currently	1.11 (0.28-4.42)	.879	2.06 (0.61-6.98)	.246
Yes, currently	1.13 (0.39-3.25)	.818	**3.16 (1.16-8.64)**	.025
**Physical activity**				
Moderate or High	1		1	
Low	**3.34 (1.80-6.20)**	<.001	1.66 (0.94-2.92)	.082

**Mediterranean diet**				
Low adherence, 0-5	1.93 (0.72-5.18)	.192	0.72 (0.17-2.95)	.645
Medium adherence, 6-10	1.01 (0.49-2.09)	.985	1.45 (0.74-2.85)	.278
High adherence ≥11	1		1	
**Polypharmacy**				
No	1		1	
Yes	1.46 (0.56-3.81)	0.441	1.15 (0.54-2.41)	0.722
**CKD**				
No	1		1	
Yes	2.15 (0.66-6.97)	0.202	1.05 (0.34-3.20)	0.937
**Retinopathy**				
No	1		1	
Yes	0.48 (0.11-2.03)	0.316	0.68 (0.15-3.09)	0.612
**Neuropathy**				
No	1		1	
Yes	1.14 (0.30-4.31)	0.845	0.86 (0.16-4.52)	0.857
**CVD**				
No	1		1	
Yes	2.32 (0.84-6.43)	0.106	2.69 (0.85-8.45)	0.091
**Microalbuminuria**				
No	1		1	
Yes	1.27 (0.55-2.96)	0.578	0.37 (0.12-1.17)	0.089
**Mood disorders**				
No	1		1	
Yes	**2.73 (1.13-6.64)**	0.026	1.81 (0.94-3.50)	0.077
**Sulfonylureas**				
Never	1		1	
Yes, but not currently	0.54 (0.11-2.76)	0.460	4.05 (0.95-17.2)	0.059
Yes, currently	0.92 (0.21-3.97)	0.909	1.05 (0.31-3.55)	0.943
**DPP4-inhibitors**				
Never	1		1	
Yes, but not currently	1.34 (0.37-4.81)	0.656	0.51 (0.11-2.44)	0.395
Yes, currently	0.62 (0.26-1.49)	0.284	1.48 (0.67-3.25)	0.332
**SGLT2-inhibitors**				
Never	1		1	
Yes, but not currently	0.96 (0.18-5.11)	0.966	0.27 (0.04-2.07)	0.209
Yes, currently	0.56 (0.25-1.25)	0.158	1.04 (0.52-2.08)	0.905
**Insulin**				
Never	1		1	
Yes, but not currently	0.65 (0.17-2.57)	0.542	0.95 (0.33-2.72)	0.921
Yes, currently	1.02 (0.40-2.61)	0.969	0.46 (0.13-1.62)	0.459
**Mood disorder drugs**				
Never	1		1	
Yes, but not currently	0.92 (0.25-3.40)	0.897	0.92 (0.32-2.66)	0.880
Yes, currently	1.01 (0.29-3.41)	0.999	1.70 (0.74-3.88)	0.210
**Antiplatelet drugs**				
Never	1		1	
Yes, but not currently	1.11 (0.27-4.49)	0.886	1.19 (0.32-4.38)	0.795
Yes, currently	1.17 (0.40-3.41)	0.775	2.42 (0.83-7.00)	0.104
